# Clinical value of coagulation parameters in predicting the severity of severe fever with thrombocytopenia syndrome

**DOI:** 10.3389/fmicb.2024.1335664

**Published:** 2024-04-03

**Authors:** Yanyan Xia, Bei Jia, Yuxin Chen, Sen Wang, Xuejing Xu

**Affiliations:** ^1^Department of Clinical Laboratory Medicine, Nanjing Drum Tower Hospital, Affiliated Hospital of Medical School, Nanjing University, Nanjing, China; ^2^Department of Infectious Diseases, Nanjing Drum Tower Hospital, Affiliated Hospital of Medical School, Nanjing University, Nanjing, China

**Keywords:** novel bunya virus, severe fever with thrombocytopenia syndrome, coagulation function, coagulation factors, D-Dimer

## Abstract

**Introduction:**

Severe fever with thrombocytopenia syndrome (SFTS) is an emerging infectious disease caused by a novel bunyavirus infection with a high lethality rate. The purpose of this study was to investigate the changes in coagulation parameters in patients with SFTS, aiming to provide clinical evidence for early diagnosis, treatment, and disease analysis.

**Methods:**

A total of 40 patients with SFTS attended from April 1, 2020 to May 21, 2022 in Nanjing Drum Tower Hospital were selected and grouped according to the duration of the disease, mild and severe disease, cure and death, with 50 healthy physical examiners as controls, and the risk of severe and death disease was predicted using ROC curves.

**Results:**

Comparison between the healthy, mild and severe groups revealed that PT, INR, APTT, TT, D-D and vWF levels were higher than those in the healthy control group, and FII, FIX, FX, FXI, FXII, PC and PS levels were lower than those in the healthy control group, the differences were statistically significant (*p* < 0.05). Comparing the results of SFTS patients with different course times, the results of Fib, FV, FVII, FVIII, FIX, FX, FXI were statistically significant (*p* < 0.05). Among the survived and deceased patients, the PT, INR, DD and PS results of the deceased patients were higher than those of the survived patients, and the FVIII, FIX, FXI, FXII and PC were lower than those of the survived patients. The area under the ROC curve showed that D-D had higher predictive ability for the risk of severe disease (AUROC 0.93, sensitivity and specificity at a Cut-off value of 1.50 mg/L were 90.0 and 86.5%, respectively) and the risk of death occurring (AUROC 0.84, sensitivity and specificity at a Cut-off value of 3.39 mg/L were 87.5 and 80.0%, respectively).

**Discussion:**

The monitoring of the coagulation parameters in patients with SFTS is great significance for identifying the severity and death of the patient’s condition, and it is of great clinical value to provide early attention, timely intervention and maximum reduction of the mortality rate for patients at risk of severe disease.

## 1 Introduction

Severe fever with thrombocytopenia syndrome (SFTS) is a newly discovered infectious disease in China, caused by the novel bunya virus infection (Dou et al., 2021; Kirino et al., 2021; Miao et al., 2021; Zhang et al., 2022). SFTS has a rapid onset with nonspecific early clinical manifestations, often presenting as fever of unknown origin and fatigue. Laboratory tests commonly reveal thrombocytopenia and leukopenia, while severe cases can lead to coma, shock, systemic diffuse vascular bleeding, and multiple organ dysfunction, resulting in high mortality rates and posing significant health threats and serious economic burdens to patients (Jiang et al., 2021; Park et al., 2021; Tsuru et al., 2021; Jeon et al., 2023). The reported cases of this infectious disease have been increasing both domestically and internationally, due to the complex pathogenesis, diverse clinical presentations, and lack of specificity, patients are often diagnosed with varying degrees of organ damage, and severe cases progress rapidly with a mortality rate ranging from 12 to 30% (Hu et al., 2021; Yoo et al., 2021; Yuan et al., 2021). Recognizing the high lethality and potential for pandemic transmission, SFTS is classified as a nationally reported disease in China, and the World Health Organization (WHO) listed SFTS as one of the top 10 priority infectious diseases in urgent need of investigation in 2017 (WHO R&D Blueprint, 2017). Therefore, early identification and assessment of risk factors in the early stages of the disease aim to determine the severity of the patient’s condition, improve the cure rate for critically ill patients, provide a theoretical basis for clinical diagnosis and treatment, and become a focus of attention for both clinical and medical laboratory work.

This study analyzed the results of coagulation function and coagulation factor, including prothrombin time (PT), international normalized ratio (INR), activated partial thromboplastin time (APTT), thrombin time (TT), fibrinogen (FIB), D-Dimer (D-D), coagulation factorII (FII), FV, FVII, FVIII, FIX, FX, FXI, FXII, protein C (PC), protein S (PS), von willebrand factor (vWF) activity, in SFTS patients at different stages, clinical outcomes, severity levels, and in healthy individuals. With the aim of identifying the severity of SFTS in patients and the probability of developing severe illness and mortality, providing early diagnosis and striving for effective symptomatic supportive treatment holds great clinical value in reducing the mortality rate for patients at risk of severe disease.

## 2 Information and methods

### 2.1 Ethical statement

The study protocol was approved by the Affiliated Drum Tower Hospital of Nanjing University Medical School (Ethics code: 2022-238-02). All patients signed written informed consent.

### 2.2 Research object

From April 1, 2020 to May 21, 2022, 40 patients of SFTS diagnosed in the affiliated Drum Tower Hospital of Nanjing University Medical School were selected. The diagnosis met the diagnostic criteria of the Severe fever with thrombocytopenia syndrome guidelines (2010 edition). Clinical data and laboratory test indexes of these patients were collected. Inclusion criteria were as follows: (1) availability of epidemiological data, (2) presence of thrombocytopenia, (3) fever with a temperature greater than 37.5°C, and (4) positive SFTSV nucleic acid test and IgG or IgM antibody. Exclusion criteria were as follows: (1) patients with concomitant viral infections, (2) patients with hematological disorders such as leukemia and idiopathic thrombocytopenic purpura, (3) patients with autoimmune diseases, (4) patients who received blood transfusions within two weeks, and (5) patients with cancer undergoing radiotherapy or chemotherapy.

The course of the disease was categorized into three stages based on the time of symptom onset, which is described by the patient. Stage I (0 ~ 7 days), stage II (8 ~ 14 days), and stage III (>14 days).

The severe group was defined by the presence of any of the following conditions: shock, coma, renal failure, liver failure, heart failure, sepsis, upper gastrointestinal bleeding, respiratory distress syndrome, impaired consciousness, DIC, etc., while all other cases were included in the mild group.

In addition, 50 healthy physical examiners were selected as controls, and written informed consent has been obtained from all patients.

### 2.3 Sample collection and processing

3 mL of fasting peripheral venous blood were collected from SFTS patients in the early morning. The blood was thoroughly mixed with 109 mmol/L sodium citrate at a ratio of 9:1. The plasma was separated by centrifugation at 1500 × g for 15 min and immediately tested or stored at −20°C for further analysis. All plasma samples were stored at −20°C for no more than one week to avoid repeated freeze–thaw cycles.

### 2.4 Laboratory measurements

#### 2.4.1 Coagulation function tests

PT, APTT, and TT were performed using coagulation assays to observe the time of fibrin clot formation. Fib levels were determined using the Clauss method, which establishes the clotting time of diluted plasma as inversely proportional to the concentration of plasma fibrinogen. D-D levels were measured using an immunoturbidimetric method to observe changes in turbidity. The measurements were conducted using the Sysmex CS-5100 automated coagulation analyzer.

#### 2.4.2 Coagulation factor tests

Coagulation factors FII, FV, FVII, FVIII, FIX, FX, FXI, FXII, PC, PS and vWF activity were measured using the Sysmex coagulation factor activity assay kits on the Sysmex CS-5100 automated coagulation analyzer at Nanjing Drum Tower Hospital.

#### 2.4.3 Biochemical indicators tests

C-reactive Protein (CRP), Lactate Dehydrogenase (LDH), Creatine Kinase (CK), Alanine Aminotransferase (ALT), Aspartate Aminotransferase (AST), Albumin (ALB), γ-Glutamyltransferase (GGT), Alkaline Phosphatase (ALP), UREA and Creatinine (CREA) were measured using Beckman automatic biochemical analyzer at Nanjing Drum Tower Hospital.

#### 2.4.4 Platelet (PLT) test

PLT was measured using Sysmex XN automatic blood analyzer by electrical impedance spectroscopy at Nanjing Drum Tower Hospital.

Strict quality control was ensured before, during, and after the analysis to guarantee the accuracy of the experimental results.

### 2.5 Statistical analysis

The statistical software SPSS 21.0 and GraphPad Prism 8.0.2 were used to analyze the data. Normally distributed continuous variables were expressed as mean ± standard deviation ( $\bar{x}\pm s$ ). Independent sample *t*-tests were used for comparisons between two groups, and one-way ANOVA was used for comparisons among multiple groups. Categorical variables were expressed as percentages (*n*, %) and analyzed using the *X^2^* test or Fisher’s exact test. Receiver operating characteristic (ROC) curves were used to evaluate the sensitivity and specificity of coagulation parameters in predicting the risk of developing severe illness and mortality in SFTS patients. Correlations were analyzed using Spearman’s correlation coefficient. The difference of *p* < 0.05 was statistically significant.

## 3 Results

### 3.1 Demographic and clinical characteristics of SFTS patients

A total of 40 patients with SFTS attended from April 1, 2020 to May 21, 2022 in Nanjing Drum Tower Hospital were selected. Among them, 29 patients survived (72.5%), while 11 patients deceased (27.5%). The average age of the patients was 61.07 ± 14.87 years, with the deceased group (72.75 ± 9.26 years) being older than the survived group (58.47 ± 14.64 years). The length of Hospitalization was shorter for the deceased group (2.75 ± 2.68 days) compared to the survived group (10.25 ± 4.06 days). The incidence of abdominal pain was higher in the deceased group (27.3%) than in the survived group (3.4%), while the incidence of shiver was lower in the deceased group (0.0%) compared to the survived group (31.0%). No significant differences were observed in the remaining indicators between the two groups (Table 1).

**Table 1:** Demographic and clinical characteristics of SFTS patients.

Clinical characteristics	Total (*n* = 40)	Survived (*n* = 29)	Deceased (*n* = 11)	t/X^2^ value	*p* value
Age (years)	61.07 ± 14.87	58.47 ± 14.64	72.75 ± 9.26	3.002	0.005
Male, *n* (%)	24/40	18/29	6/11	0.188	0.665
Time from onset to admission (days)	7.96 ± 2.76	8.14 ± 2.66	7.00 ± 3.08	1.159	0.254
Hospitalization (days)	9.00 ± 4.77	10.25 ± 4.06	2.75 ± 2.68	5.653	0.000
Highest body temperature (°C)	38.51 ± 0.74	38.49 ± 0.80	38.65 ± 0.27	0.645	0.523
History, *n* (%)					
Hypertensive disease, *n* (%)	9/40 (22.5%)	8/29 (27.6%)	1/11 (9.1%)	1.564	0.211
Diabetes, *n* (%)	2/40 (5.0%)	2/29 (6.9%)	0/11 (0.0%)	0.799	0.372
Cerebral infarction, *n* (%)	4/40 (10.0%)	2/29 (6.9%)	2/11 (18.2%)	1.129	0.288
Coronary heart disease, *n* (%)	2/40 (5.0%)	1/29 (3.4%)	1/11 (9.1%)	0.535	0.465
Liver disease, *n* (%)	12/40 (30.0%)	8/29 (27.6%)	4/11 (36.4%)	0.293	0.589
Pulmonary diseases, *n* (%)	13/40 (32.5%)	10/29 (34.5%)	3/11 (27.3%)	0.189	0.664
kidney disease, *n* (%)	6/40 (15.0%)	3/29 (10.3%)	3/11 (27.3%)	1.792	0.181
Tumor related history, *n* (%)	2/40 (5.0%)	2/29 (6.9%)	0/11 (0.0%)	0.799	0.372
Symptoms					
Weak, *n* (%)	17/40 (42.5%)	13/29 (44.8%)	4/11 (36.4%)	0.234	0.629
Inappetence, *n* (%)	15/40 (37.5%)	11/29 (37.9%)	4/11 (36.4%)	0.008	0.927
Diarrhea, *n* (%)	5/40 (12.5%)	3/29 (10.3%)	2/11 (18.2%)	0.448	0.503
Abdominal pain, *n* (%)	4/40 (10.0%)	1/29 (3.4%)	3/11 (27.3%)	5.03	0.025
Nausea, *n* (%)	7/40 (17.5%)	5/29 (17.2%)	2/11 (18.2%)	0.05	0.944
Vomiting, *n* (%)	6/40 (15.0%)	4/29 (13.8%)	2/11 (18.2%)	0.12	0.729
Shiver, *n* (%)	9/40 (22.5%)	9/29 (31.0%)	0/11 (0.0%)	4.405	0.036
Muscular soreness, *n* (%)	4/40 (10.0%)	4/29 (13.8%)	0/11 (0.0%)	1.686	0.194
lymphadenopathy, *n* (%)	13/40 (32.5%)	11/29 (37.9%)	2/11 (18.2%)	1.418	0.234
Neurological symptoms, *n* (%)	2/40 (5.0%)	1/29 (3.4%)	1/11 (9.1%)	0.535	0.465

### 3.2 Comparison of coagulation parameters in healthy, mild and severe groups of SFTS

**Figure 1:**
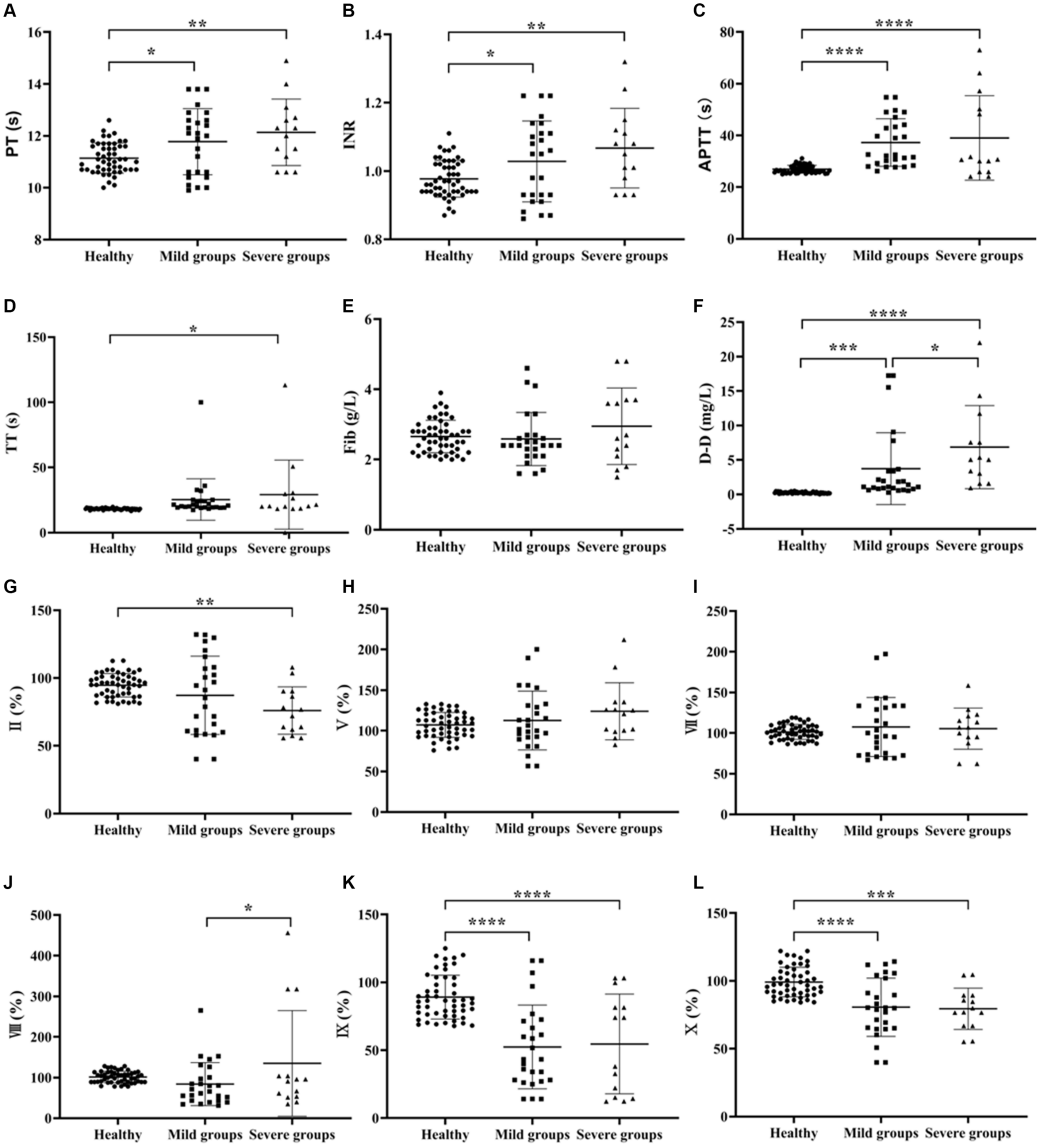

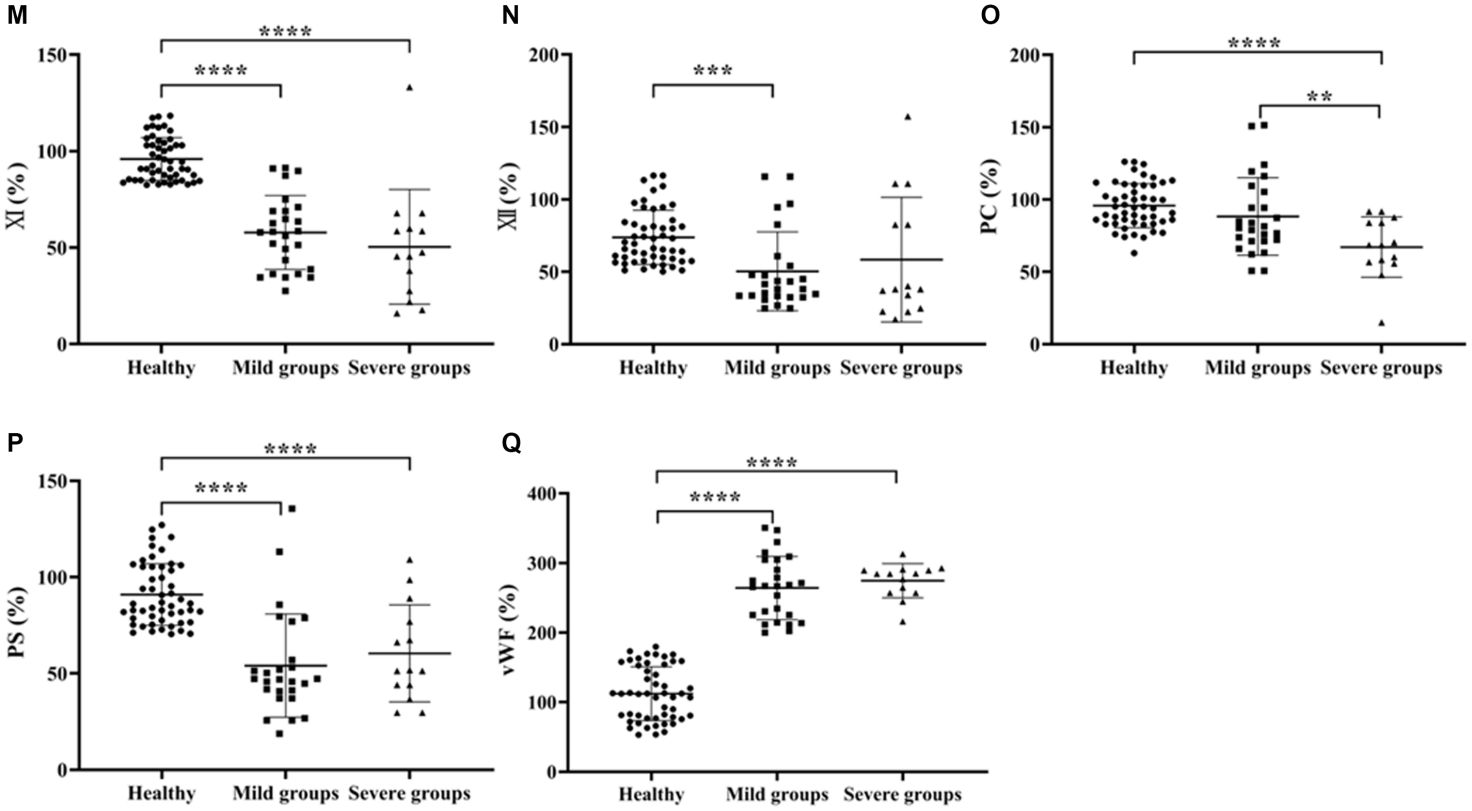
Comparison of coagulation parameters in healthy, mild and severe groups of SFTS. **(A–F)** Comparison of coagulation function, the results showed that the difference in Fib results alone was not statistically significant (*p* > 0.05), and the levels of other items such as PT, INR, APTT, TT and D-D were higher than those in the healthy control group; **(G–Q)** comparison of coagulation factor, there was no significant difference in the results of FV, FVII and FVIII (*p* > 0.05). The remaining items such as FII, FIX, FX, FXI, FXII, PC and PS levels were lower in the SFTS groups compared to the healthy control group, while the levels of vWF were higher in the SFTS groups, with statistically significant differences (*p* < 0.05).

One-way ANOVA was used to compare the Coagulation function between the healthy, mild and severe groups of SFTS. The results showed that the difference in Fib results alone was not statistically significant (*p* > 0.05), and the levels of other items such as PT, INR, APTT, TT and D-D were higher than those in the healthy control group, and the differences were statistically significant (*p* < 0.05). Among these, the elevation of D-D levels was the most significant (Figures 1A–F).

In terms of coagulation factor results, there was no significant difference in the results of FV, FVII, FVIII and FXII (*p* > 0.05). The remaining items such as FII, FIX, FX, FXI, PC and PS levels were lower in the SFTS groups compared to the healthy control group, while the levels of vWF were higher in the SFTS groups, with statistically significant differences (*p* < 0.05) (Figures 1G–Q).

### 3.3 Comparison of coagulation parameters in SFTS patients with different disease duration

**Figure 2:**
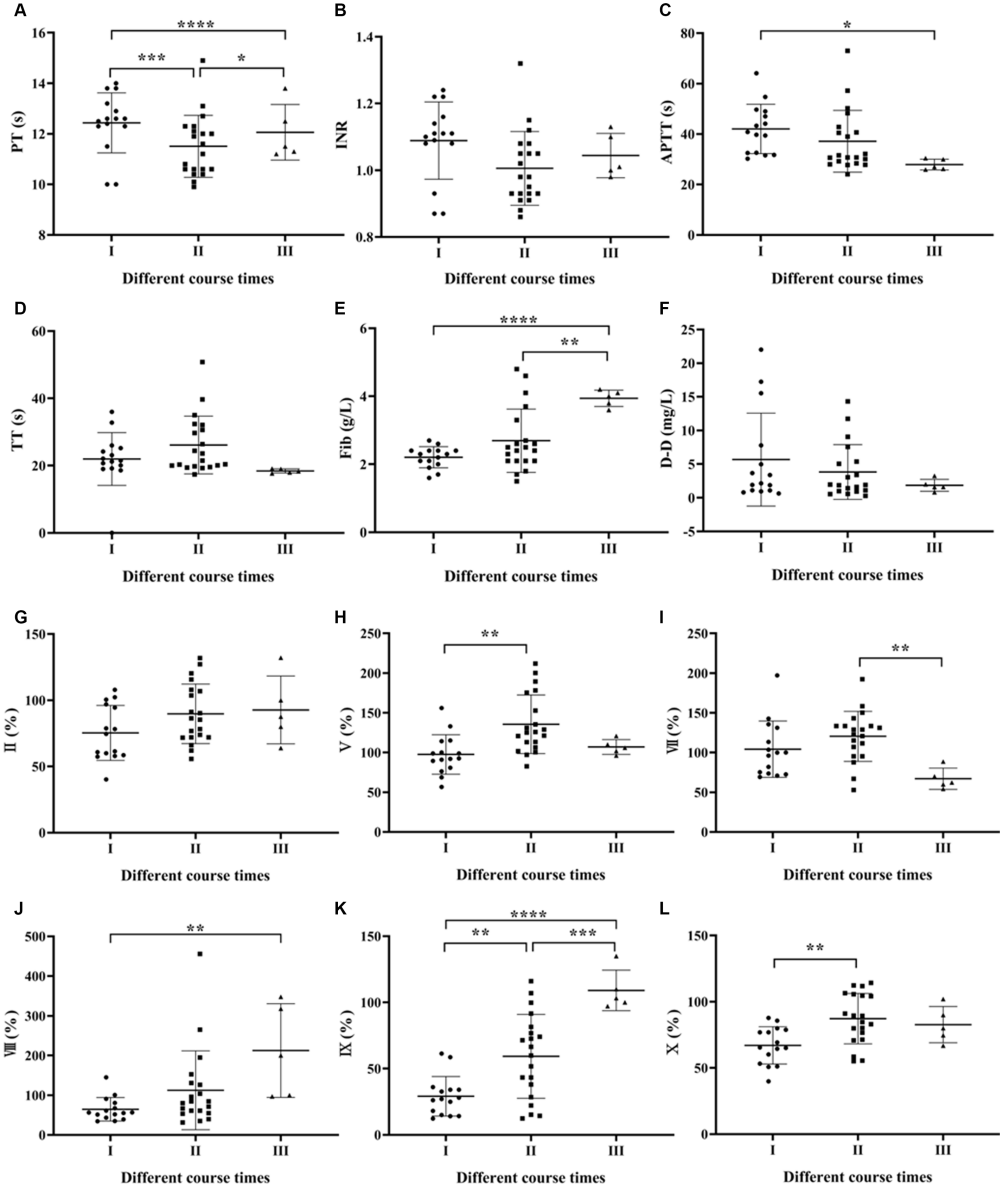

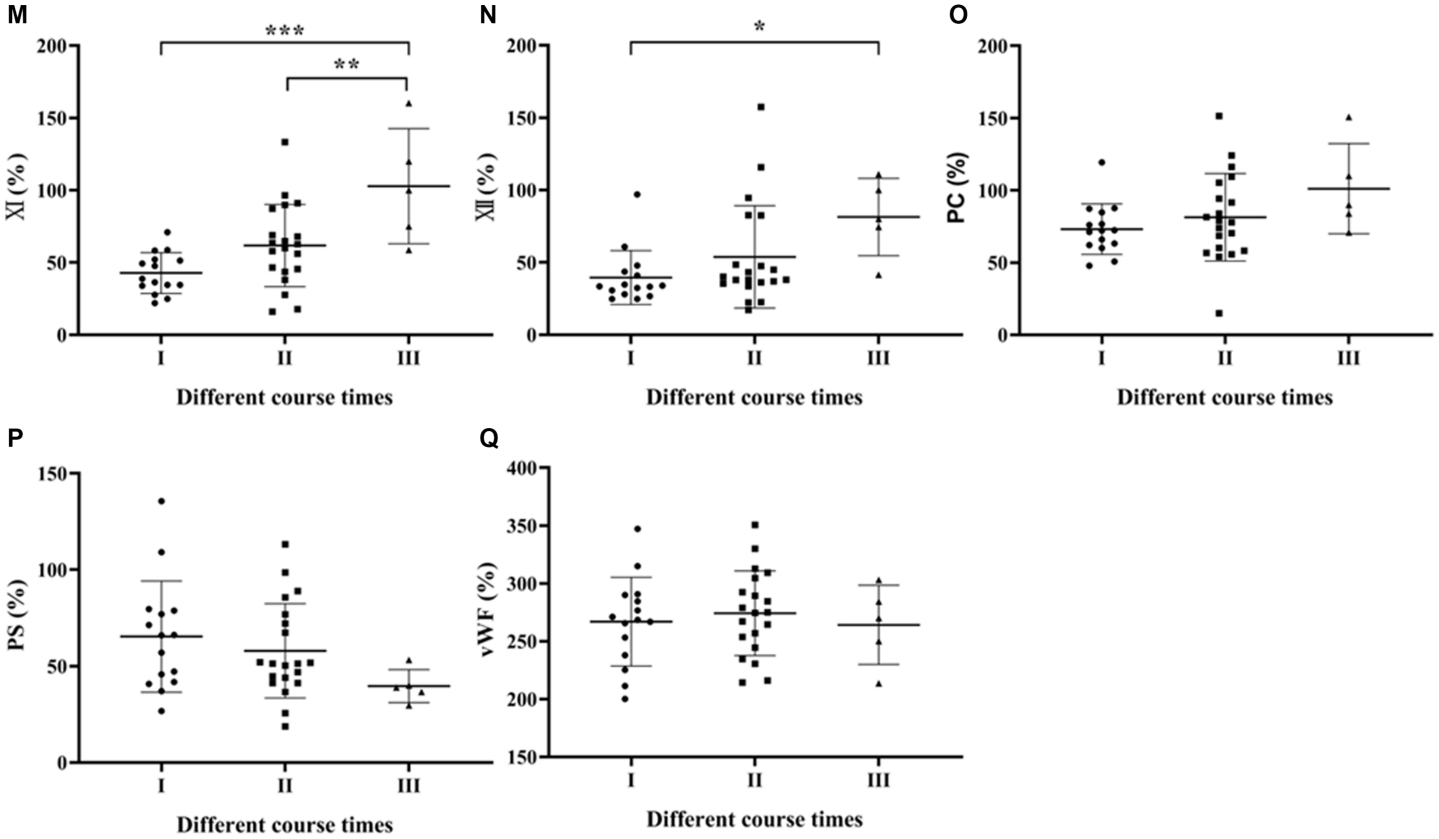
Comparison of coagulation parameters in SFTS patients with different course times. **(A–F)** Comparison of coagulation function, the results showed that only the Fib levels had a statistically significant difference (*p* < 0.05), while the remaining results were not statistically significant (*p* > 0.05); **(G–Q)** comparison of coagulation factor, the differences in factors FV, FVII, FVIII, FIX, FX, FXI, and FXI were statistically significant (*p* < 0.05).

One-way ANOVA was used to compare the coagulation parameters among SFTS patients with different disease duration. The results showed that only the APTT and Fib levels had a statistically significant difference (*p* < 0.05), while the remaining results were not statistically significant (*p* > 0.05) (Figures 2A–F).

Regarding coagulation factor results among SFTS patients with different disease duration, the differences in factors FV, FVII, FVIII, FIX, FX, FXI and FXII were statistically significant (*p* < 0.05) (Figures 2G–Q).

### 3.4 Comparison of coagulation parameters results between patients with survived and deceased SFTS

**Figure 3:**
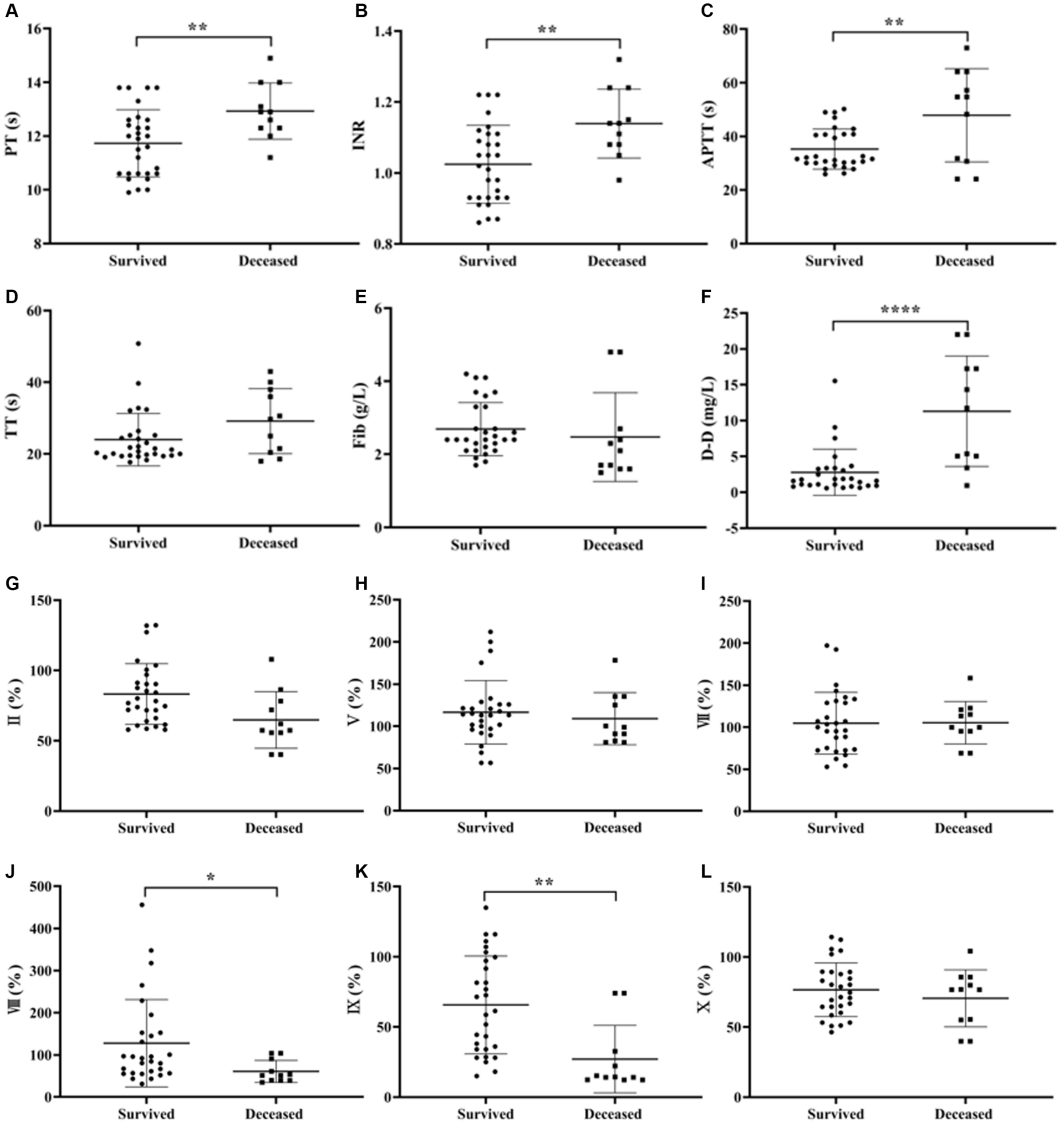

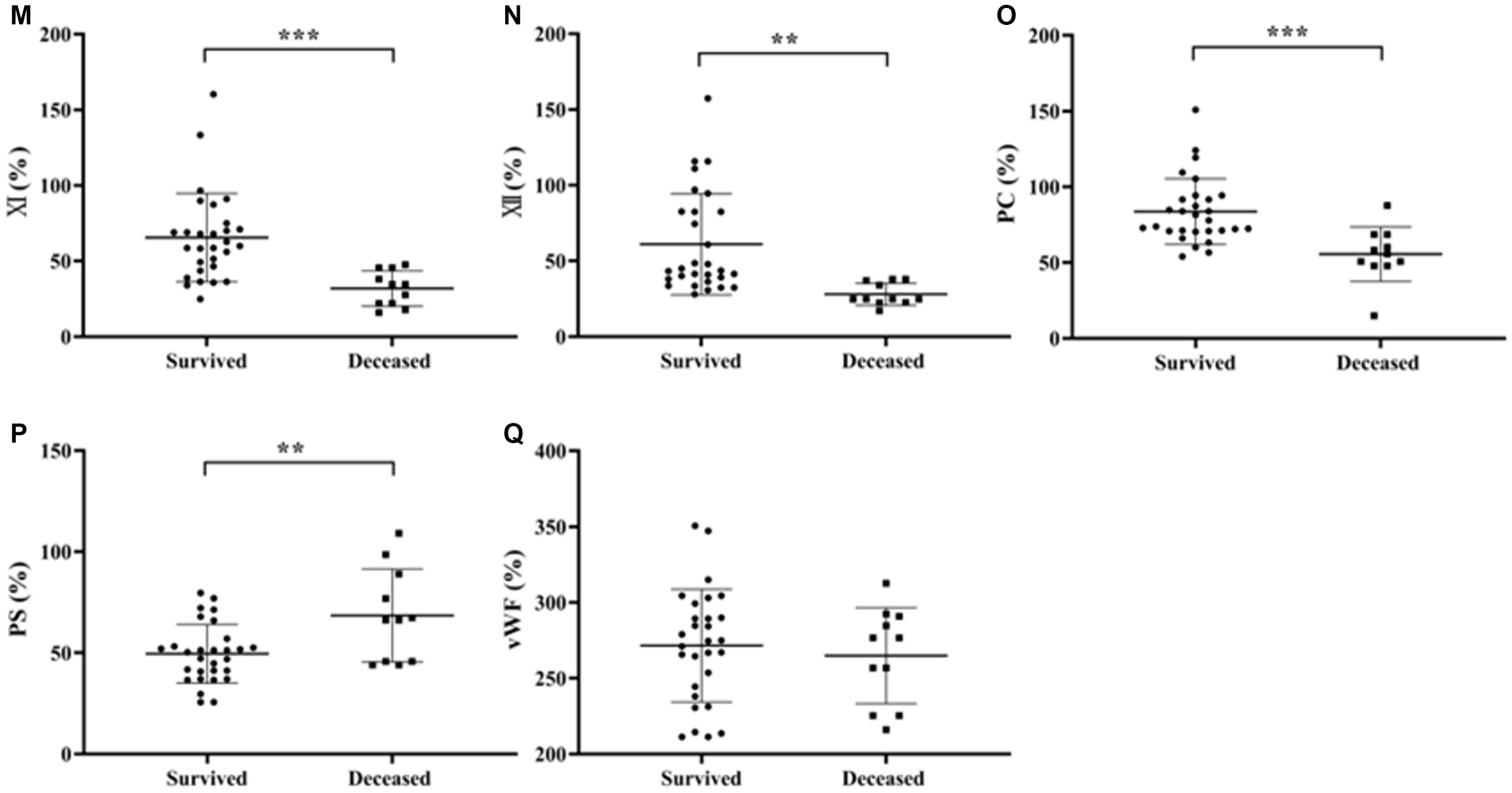
Comparison of coagulation parameters results between patients with survived and deceased SFTS. **(A–F)** Comparison of coagulation function, the results showed that the PT, INR, and D-D levels were higher in deceased patients compared to survived patients, and the differences were statistically significant (*p* < 0.05); **(G–Q)** comparison of coagulation factor, deceased patients had lower levels of FVIII, FIX, FXI, FXII and PC, while the level of PS was higher in deceased patients compared to survived patients, and these differences were statistically significant (*p* < 0.05).

Independent samples t-test was used to analyze the differences in coagulation parameters between survived and deceased SFTS patients. The results showed that the PT, INR, APTT and D-D levels were higher in deceased patients compared to survived patients, and the differences were statistically significant (*p* < 0.05) (Figures 3A–F).

Regarding coagulation factor results between survived and deceased SFTS patients (Figures 3G–Q), deceased patients had lower levels of FVIII, FIX, FXI, FXII and PC, while the level of PS was higher in deceased patients compared to survived patients, and these differences were statistically significant (*p* < 0.05).

### 3.5 ROC analysis for predicting disease severity and mortality risk in SFTS patients

**Figure 4:**
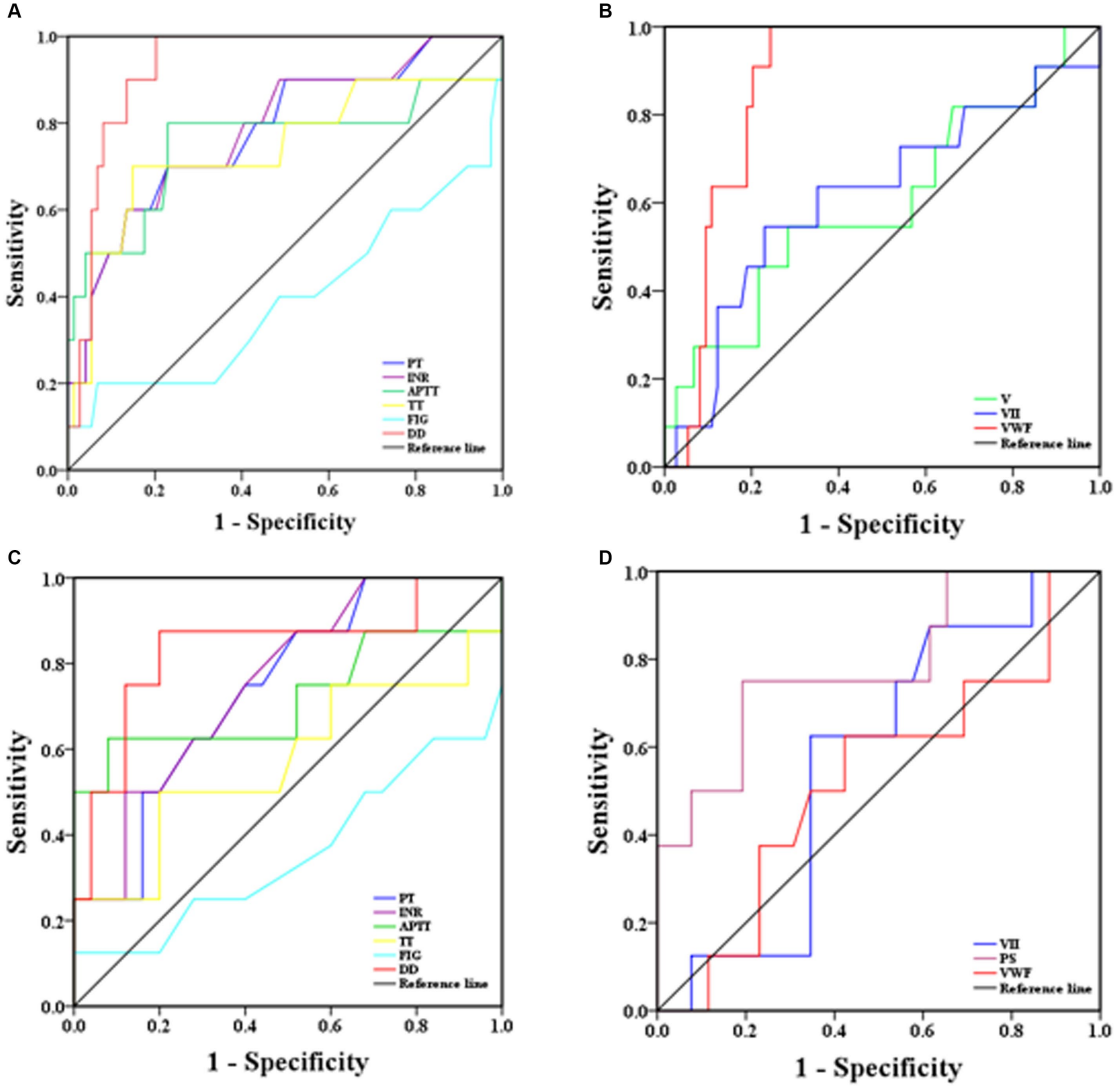
ROC analysis of coagulation parameters for predicting severity and mortality risk in SFTS patients [**(A)** coagulation routine predicting disease severity, **(B)** coagulation factor results predicting disease severity, **(C)** coagulation routine predicting mortality risk, **(D)** coagulation factor results predicting mortality risk].

ROC curves were used to determine the sensitivity and specificity of coagulation parameters in predicting the risk of severe and mortality illness in SFTS patients, and the area under the ROC curve (AUROC) was used to assess their predictive ability. Generally, AUROC >0.90 indicates high predictive ability, AUROC between 0.70 and 0.90 indicates moderate predictive ability, and AUROC <0.70 indicates low predictive ability. From the ROC curves (Figure 4A), D-D had the highest predictive ability for the risk of severe illness, with an AUROC of 0.93 (95% CI: 0.87–0.99). The sensitivity and specificity at a cut-off point of 1.50 mg/L were 90.0 and 86.5%, respectively. The predictive abilities of the PT, INR, APTT, and TT, were moderate, with AUROC values around 75%. Fib had the lowest predictive ability, with an AUROC of only 39% (Figure 4A). In terms of coagulation factor prediction for severe illness, vWF had the highest predictive ability, with an AUROC of 0.87 (95% CI: 0.80–0.95). The sensitivity and specificity at a cut-off point of 255.0% were 81.8 and 81.1%, respectively. The AUROC values for the remaining factors were all <0.70, indicating low predictive ability (Figure 4B).

ROC curves were also used to predict the mortality risk in SFTS patients. D-D had the highest predictive ability, with an AUROC of 0.84 (95% CI: 0.65–0.99). The sensitivity and specificity at a cut-off point of 3.39 mg/L were 87.5 and 80.0%, respectively. The predictive abilities of PT, INR, and APTT were moderate, with AUROC values around 73.0%. TT and Fib had the lowest predictive abilities, with AUROC values around 40.0% (Figure 4C). Regarding coagulation factor prediction for mortality risk, PS had the highest predictive ability, with an AUROC of 0.78 (95% CI: 0.80–0.95). The sensitivity and specificity at a cut-off point of 66.1% were 75.0 and 80.8%, respectively. The AUROC values for the remaining factors were all <0.60, indicating low predictive ability (Figure 4D).

### 3.6 Correlation analysis

**Figure 5:**
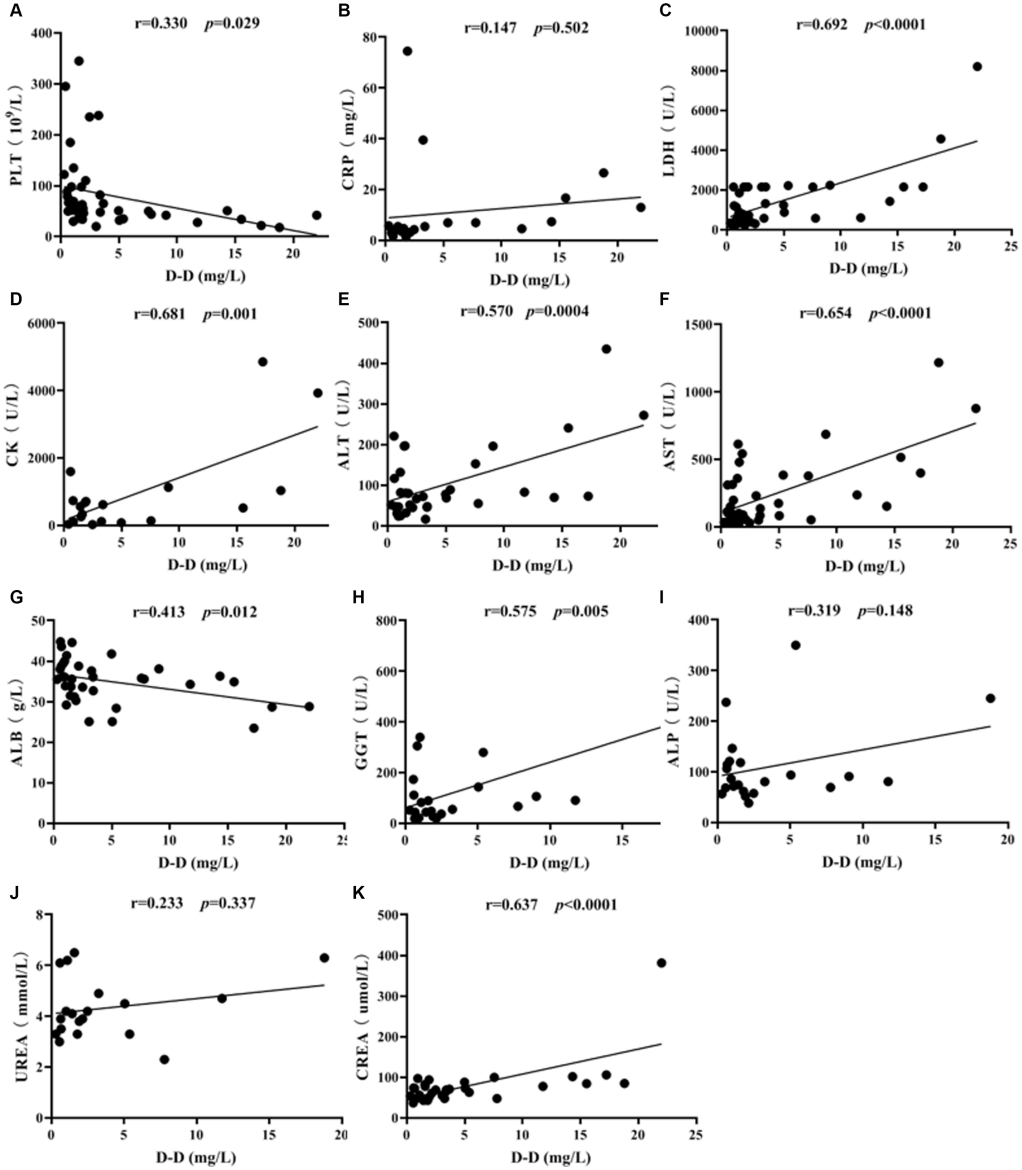
Correlation analysis of D-D levels with PLT and biochemical parameters in SFTS patients. Correlation analysis of D-D with PLT, CRP, LDH, CK, ALT, AST, ALB, GGT, ALP, UREA and CREA, respectively **(A-K)**.

The correlation of D-D levels with PLT and biochemical parameters in SFTS patients was conducted by Spearman correlation analysis. The results showed that D-D levels were positively correlated with LDH (*p* < 0.0001), CK (*p* = 0.001), ALT (*p* = 0.0004), AST (*p* < 0.0001), GGT (*p* = 0.005), and CREA (*p* < 0.0001), while they were negatively correlated with PLT (*p* = 0.029) and ALB (*p* = 0.012) (Figure 5). There was no correlation between vWF and PS with PLT and biochemical markers (*p* > 0.05).

## 4 Discussion

SFTS is an infectious disease discovered in 2010, caused by a novel Bunyavirus, with diverse clinical manifestations and lack of specificity (Dualis et al., 2021; Chaudhuri et al., 2022; Zhu et al., 2022). When neurological and psychiatric symptoms accompanied by multi-organ dysfunction occur, the disease progresses rapidly with a poor prognosis (Wu et al., 2016; Oh et al., 2017; Li et al., 2021; Tao et al., 2021). Therefore, identifying laboratory differences between ordinary and critically ill patients and promptly providing antimicrobial and immune-enhancing therapies can significantly improve patient survival rates.

This study found that the average age of deceased patients was higher than that of survived patients. Previous research has also shown that SFTS is more common in middle-aged and elderly individuals over 50 years old, with no reported cases among preschool children. Advanced age is associated with lower tolerance and a worse prognosis in SFTS patients, thus contributing to higher mortality rates. This conclusion is consistent with the findings of Liu et al. (2022).

Infection with the novel bunyavirus can lead to coagulation dysfunction (Zhang et al., 2012; Paessler and Walker, 2013). In terms of coagulation parameters measured in this study, levels of PT, INR, APTT, TT, and D-D were all higher in SFTS patients compared to the healthy control group. Although PT and INR levels were higher than those in the control group, they were within the normal reference range and had limited clinical significance. On the other hand, APTT, TT, and D-D levels gradually increased with disease progression, indicating their elevation correlated with the severity of the disease. Analysis of coagulation factor results revealed lower levels of FII, FIX, FX, FXI, FXII, PC, PS, and higher levels of vWF in SFTS patients compared to the healthy control group. The deficiency of coagulation factors may be attributed to chronic activation of endothelial cells caused by systemic inflammation, leading to impaired secretion of coagulation factors and increased consumption (Panin et al., 2021; André et al., 2022; Modrzycka et al., 2022). Moreover, increased plasma vWF levels can be observed when endothelial cells are stimulated or damaged and the body is under stress (Jeong et al., 2022; Rayner et al., 2022).

Regarding the duration of illness, the study observed a gradual increase in Fib levels with longer duration. Although Fib, as an acute-phase reactant protein, increases in various diseases such as infection and tissue damage and can reflect the coagulation status of the body (Xu et al., 2021), the elevated Fib levels in this study remained within the normal reference range and had limited clinical significance. Among the coagulation factor results, statistically significant differences were found in levels of FV, FVII, FVIII, FIX, FX and FXI, with the lowest levels observed in Phase I and gradual recovery to normal levels in Phases II and III.

In comparisons between survived and deceased patients, deceased patients had higher PT, INR, and D-D levels than survived patients, with D-D showing the most significant difference. Among the coagulation factor results, deceased patients had lower levels of FVIII, FIX, FXI, FXII and PC, while they had higher levels of PS compared to survived patients. The decreased levels of endogenous coagulation factors in deceased patients indicate a higher risk of bleeding.

ROC curve analysis was conducted to assess the predictive ability of coagulation parameters for the risk of severe illness and mortality in SFTS patients. The results revealed that D-D had the highest predictive ability for severe illness risk, with an AUROC of 0.93 (95% CI: 0.87–0.99), and the sensitivity and specificity were 90.0 and 86.0%, respectively. The second-highest predictive ability was observed for vWF, with an AUROC of 0.87 (95% CI: 0.80–0.95), and the sensitivity and specificity were 82.0 and 81.0%, respectively. The remaining parameters had lower predictive abilities, suggesting that timely treatment should be administered when D-D and vWF levels are abnormally elevated to prevent disease progression to a severe state, which poses a significant risk to the patient’s life. Regarding the prediction of mortality risk in SFTS patients, D-D had the highest predictive ability, with an AUROC of 0.84 (95% CI: 0.65–0.99), and the sensitivity and specificity were 87.5 and 80.0%, respectively. The second-highest predictive ability was observed for PS, with an AUROC of 0.78 (95% CI: 0.80–0.95), and the sensitivity and specificity were 75.0 and 80.8%, respectively. These findings suggest that increased D-D and PS levels are associated with an elevated risk of mortality, emphasizing the need for timely and effective interventions.

Correlation analysis revealed that D-D levels were positively correlated with LDH (*p* < 0.0001), CK (*p* = 0.001), ALT (*p* = 0.0004), AST (*p* < 0.0001), GGT (*p* = 0.005), and CREA (*p* < 0.0001). Additionally, AST levels were higher than ALT levels, indicating severe liver cell damage. Significant elevations in LDH and CK suggest damage to both cardiac and skeletal muscle cells, indicating the broad tropism of SFTSV for organs such as the heart, liver, and kidneys. D-D levels were negatively correlated with PLT (*p* = 0.029) and ALB (*p* = 0.012), indicating that as the severity of SFTS increases, PLT levels decrease, and liver damage becomes more pronounced, resulting in lower ALB levels.

## 5 Conclusion

In summary, this study not only tested routine coagulation indicators such as PT, APTT, TT, and D-dimer but also systematically and comprehensively analyzed the changes in 11 coagulation factors in SFTS patients. It was found that D-D has the most significant predictive ability for the risk of severe illness and mortality, followed by vWF, which also shows strong predictive power for the risk of severe illness, and PS, which similarly exhibits a high predictive capacity for the risk of death. Therefore, closely monitoring these coagulation parameters is critically important for tracking and assessing the progression of the disease in SFTS patients. In addition, it is crucial to be familiar with the clinical characteristics and laboratory fluctuations in critically ill cases, aiming to achieve early detection, diagnosis, and effective supportive treatment to minimize the mortality rate. However, this study is limited by its sample size, further large-scale, multicenter, high-quality randomized controlled trials are needed to provide more reliable application guidelines for clinical practice.

## Data availability statement

The original contributions presented in the study are included in the article/supplementary material, further inquiries can be directed to the corresponding authors.

## Ethics statement

The studies involving humans were approved by the Affiliated Drum Tower Hospital of Nanjing University Medical School (Ethics code: 2022-238-02). The studies were conducted in accordance with the local legislation and institutional requirements. The participants provided their written informed consent to participate in this study.

## Author contributions

YX: Methodology, Writing – original draft. BJ: Conceptualization, Writing – review & editing. YC: Conceptualization, Data curation, Writing – review & editing. SW: Funding acquisition, Investigation, Methodology, Writing – review & editing. XX: Data curation, Formal analysis, Writing – review & editing.

## References

[ref1] André L. L. Mina N. Bonturi C. R. Nogueira R. S. RJS T. MLV O.. (2022). Anionic Ultrasmall gold nanoparticles bind to coagulation factors and disturb Normal hemostatic balance. Chem. Res. Toxicol. 35, 1558–1569. doi: 10.1021/acs.chemrestox.2c00190 36018252

[ref2] Chaudhuri D. Datta J. Majumder S. Giri K. (2022). In silico study on mi RNA regulation and NSs protein interactome characterization of the SFTS virus. J. Mol. Graph. Model. 117:108291. doi: 10.1016/j.jmgm.2022.108291, PMID: 35977432

[ref3] Dou L. Tao X. Zhao W. Zheng G. Lu Y. Tong W.. (2021). shRNA targeting nonstructural protein inhibits the replication of severe fever with thrombocytopenia syndrome virus. Future Virol. 16, 451–458. doi: 10.2217/fvl-2020-0186

[ref4] Dualis H. Zefong A. C. Joo L. K. Dadar Singh N. K. Syed Abdul Rahim S. S. Avoi R.. (2021). Factors and outcomes in severe fever with thrombocytopenia syndrome (SFTS): a systematic review. Ann. Med. Surg. 67, 102501–102506. doi: 10.1016/j.amsu.2021.102501, PMID: 34188913 PMC8219640

[ref5] Hu L. Li J. Zhang H. Bian T. Pan J. Li J.. (2021). Predisposing factors for person-to-person transmission of severe fever with thrombocytopenia syndrome bunyavirus. J. Hosp. Infect. 123, 174–178. doi: 10.1016/j.jhin.2021.10.023 34767872

[ref20] Jeon K. Hyo-Jin R. Jun-Gu K. Da-Eun J. Joowan K. Yabeen L.. (2023). A natural variation in the RNA polymerase of severe fever with thrombocytopenia syndrome virus enhances viral replication and in vivo virulence. J. Med. Virol. 9:e29099-29110. 10.1002/jmv.29099 37702580

[ref6] Jeong H. S. Lee D. H. Kim S. H. Lee C. H. Shin H. M. Kim H. R.. (2022). Hyperglycemia-induced oxidative stress promotes tumor metastasis by upregulating vWF expression in endothelial cells through the transcription factor GATA1. Oncogene 41, 1634–1646. doi: 10.1038/s41388-022-02207-y, PMID: 35094008

[ref7] Jiang S. Shah M. K. Li C. (2021). Quantitative assessment on the epidemic characteristics of severe fever with thrombocytopenia syndrome virus infection in China. Biosaf. Health 3, 292–299. doi: 10.1016/j.bsheal.2021.07.004

[ref8] Kirino Y. Ishijima K. Miura M. Nomachi T. Mazimpaka E. Sudaryatma P. E.. (2021). Seroprevalence of severe fever with thrombocytopenia syndrome virus in small-animal veterinarians and nurses in the Japanese prefecture with the highest case load. Viruses 13, 229–234. doi: 10.3390/v13020229, PMID: 33540629 PMC7912989

[ref9] Li A. Liu L. Wu W. Liu Y. Huang X. Li C.. (2021). Molecular evolution and genetic diversity analysis of SFTS virus based on next-generation sequencing. Biosaf. Health 3, 105–115. doi: 10.1016/j.bsheal.2021.02.002

[ref10] Liu Z. Zhang R. Liu Y. Ma R. Zhang L. Zhao Z.. (2022). Eosinophils and basophils in severe fever with thrombocytopenia syndrome patients: risk factors for predicting the prognosis on admission. Plos Neglect. Trop. D 16, e0010967–e0010984. doi: 10.1371/journal.pntd.0010967, PMID: 36542604 PMC9770358

[ref11] Miao D. Liu M. J. Wang Y. X. Ren X. Lu Q. B. Zhao G. P.. (2021). Epidemiology and ecology of severe fever with thrombocytopenia syndrome in China. Clin. Infect. Dis. 73, e3851–e3858. doi: 10.1093/cid/ciaa1561, PMID: 33068430 PMC8664468

[ref12] Modrzycka S. Kołt S. Polderdijk S. G. I. Adams T. E. Potoczek S. Huntington J. A.. (2022). Parallel imaging of coagulation pathway proteases activated protein C, thrombin, and factor Xa in human plasma. Chem. Sci. 13, 6813–6829. doi: 10.1039/D2SC01108E, PMID: 35774156 PMC9200056

[ref13] Oh W. S. Yoo J. R. Kwon K. T. Kim H. I. Lee S. J. Jun J. B.. (2017). Effect of early plasma exchange on survival in patients with severe fever with thrombocytopenia syndrome: a multicenter study. Yonsei Med. J. 58, 867–871. doi: 10.3349/ymj.2017.58.4.867, PMID: 28541003 PMC5447121

[ref14] Paessler S. Walker D. H. (2013). Pathogenesis of the viral hemorrhagic fevers. Ann. Rev. Pathol. Mech. Dis. 8, 411–440. doi: 10.1146/annurev-pathol-020712-164041 23121052

[ref15] Panin M. A. Zagorodniy N. V. Boyko A. V. Samokhodskaya L. M. Abakirov M. D. Volkov E. E. (2021). The significance of polymorphisms of gene coagulation factors VII and XIII in the pathogenesis of non-traumatic avascular necrosis of the femoral head. Orthop. Genius 27, 43–47. doi: 10.18019/1028-4427-2021-27-1-43-47

[ref16] Park A. Park S. J. Jung K. L. Kim S. M. Kim E. H. Kim Y. I.. (2021). Molecular signatures of inflammatory profile and B-cell function in patients with severe fever with thrombocytopenia syndrome. MBio 12, e02583–e02520. doi: 10.1128/mBio.02583-20, PMID: 33593977 PMC8545090

[ref17] Rayner S. G. Scholl Z. Mandrycky C. J. Chen J. LaValley K. N. Leary P. J.. (2022). Endothelial-derived von Willebrand factor accelerates fibrin clotting within engineered microvessels. J. Thromb. Haemost. 20, 1627–1637. doi: 10.1111/jth.15714, PMID: 35343037 PMC10581744

[ref18] Tao L. Yi Y. Shan Y. Yu D. Zhang J. Qu Y.. (2021). Analysis on severe fever with thrombocytopenia syndrome Bunyavirus infection combined with atrial fibrillation under digital model detection. Results Phys. 26, 104413–104420. doi: 10.1016/j.rinp.2021.104413

[ref19] Tsuru M. Suzuki T. Murakami T. Matsui K. Maeda Y. Yoshikawa T.. (2021). Pathological characteristics of a patient with severe fever with thrombocytopenia syndrome (SFTS) infected with SFTS virus through a sick Cat’s bite. Viruses 13, 204–212. doi: 10.3390/v13020204, PMID: 33572914 PMC7912689

[ref21] WHO R&D Blueprint (2017). Annual review of diseases prioritized under the Research and Development blueprint (WHO Meeting report, World Health Organization R&D Blueprint). Avilable at: https://www.who.int/activities/prioritizing-diseases-for-research-and-development-in-emergency-contexts

[ref22] Wu L. Deng F. Xie Z. Hu S. Shen S. Shi J.. (2016). Spatial analysis of severe fever with thrombocytopenia syndrome virus in China using a geographically weighted logistic regression model. Int. J. Environ. Res. Public Health 13:1125. doi: 10.3390/ijerph13111125, PMID: 27845737 PMC5129335

[ref23] Xu H. Shang G. Wang Y. Xiang S. (2021). Plasma fibrinogen is a reliable marker for diagnosing periprosthetic joint infection and determining the timing of second-stage revision. Int. J. Infect. Dis. 108, 220–225. doi: 10.1016/j.ijid.2021.05.068, PMID: 34089882

[ref24] Yoo E. H. Lee A. J. Kim S. G. Jeon C. H. (2021). Therapeutic plasma exchange in patients with severe fever with thrombocytopenia syndrome: a single institution experience [J]. Korean J. Blood Transf. 32, 83–90. doi: 10.17945/kjbt.2021.32.2.83

[ref25] Yuan Y. Lu Q. Yao W. Zhao J. Zhang X. A. Cui N.. (2021). Clinical efficacy and safety evaluation of favipiravir in treating patients with severe fever with thrombocytopenia syndrome. EBioMedicine 72:103591. doi: 10.1016/j.ebiom.2021.103591, PMID: 34563924 PMC8479638

[ref26] Zhang Y. Bai X. Li J. Xie J. Li H. Yang L.. (2022). A CRISPR-based nucleic acid detection method for severe fever with thrombocytopenia syndrome virus. Virus Res. 311, 198691–198699. doi: 10.1016/j.virusres.2022.198691, PMID: 35143909

[ref27] Zhang Y. Z. He Y. W. Dai Y. A. Xiong Y. Zheng H. Zhou D. J.. (2012). Hemorrhagic fever caused by a novel Bunyavirus in China: pathogenesis and correlates of fatal outcome. Clin. Infect. Dis. 4, 527–533. doi: 10.1093/cid/cir804 22144540

[ref28] Zhu T. Cai Q. Q. Yu J. Liang X. S. (2022). Metagenomic next-generation sequencing (mNGS) confirmed a critical case of severe fever with thrombocytopenia syndrome virus (SFTSV). Clin. Chem. Lab. Med. 60, e42–e45. doi: 10.1515/cclm-2021-0791, PMID: 34674414

